# Supplementary material for: Structure of BAI1/ELMO2 complex reveals an action mechanism of adhesion GPCRs via ELMO family scaffolds
Source: Nat Commun. 2019 Jan 3;10:51. doi: 10.1038/s41467-018-07938-9 (PMC6318265; doi:10.1038/s41467-018-07938-9)
Supplement: Supplementary file 1 — Supplementary Information [file 41467_2018_7938_MOESM1_ESM.pdf]

# Supplemental Information:

**"Structure of BAI1/ELMO2 complex reveals an action mechanism of adhesion GPCRs via ELMO family scaffolds", Z. Weng et al.**

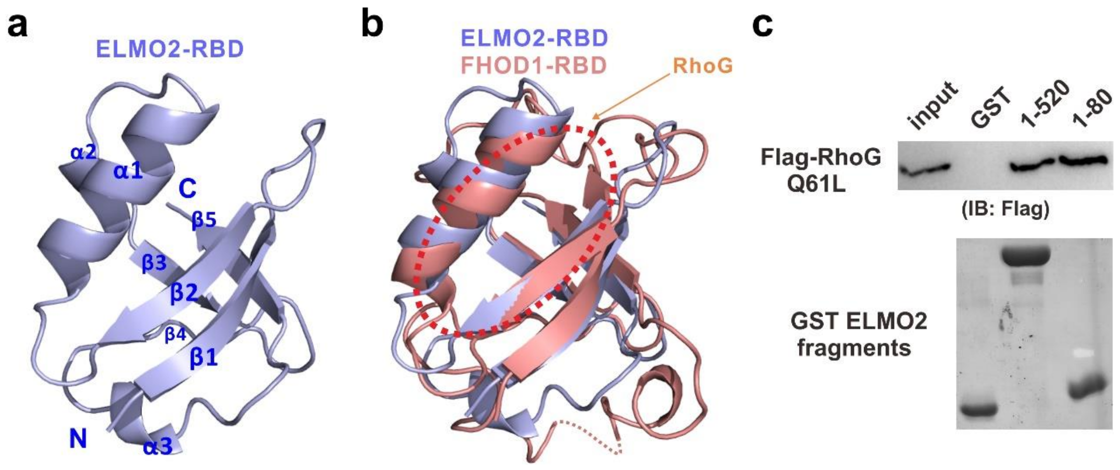


**Supplementary Figure 1 | The RBD domain is sufficient for RhoG binding. a,** Overall structure of the RBD domain of ELMO2. **b**, Superposition of the structures of ELMO2-RBD and FHOD1-RBD. Noted that the potential RhoG binding site on ELMO2-RBD is highlighted with the red dashed circle. **c**, GST-pull down assays showing that the RBD domain (aa 1-80) bound to active RhoG as effectively as the RBD-ARR-ELMO tandem (aa 1-520) did.

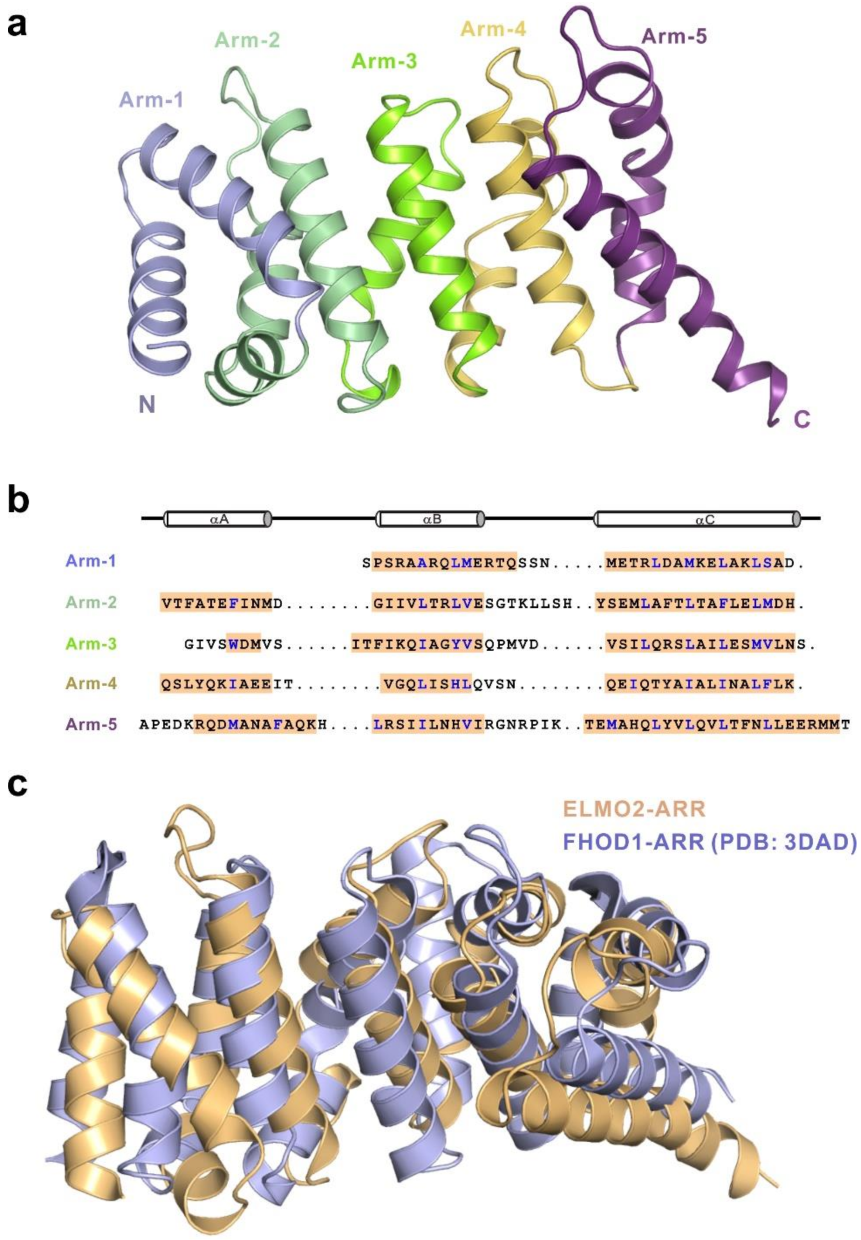


**Supplementary Figure 2 | The ARR domain of ELMO2. a,** Ribbon diagram of overall structure of the ARR domain of ELMO2. Noted that the ARM-1 is not complete, which lacking the first α-helix (i.e., ARM-1 αA). **b**, Secondary structure element comparison of the armadillo repeat structures in the ARR domain of ELMO2. α-helices are shaded orange. Hydrophobic residues that mediate the core scaffold within the armadillo repeats are marked blue. **c**, Superposition of the structures of ELMO2-ARR and FHOD1-ARR (PDB code: 3DAD).

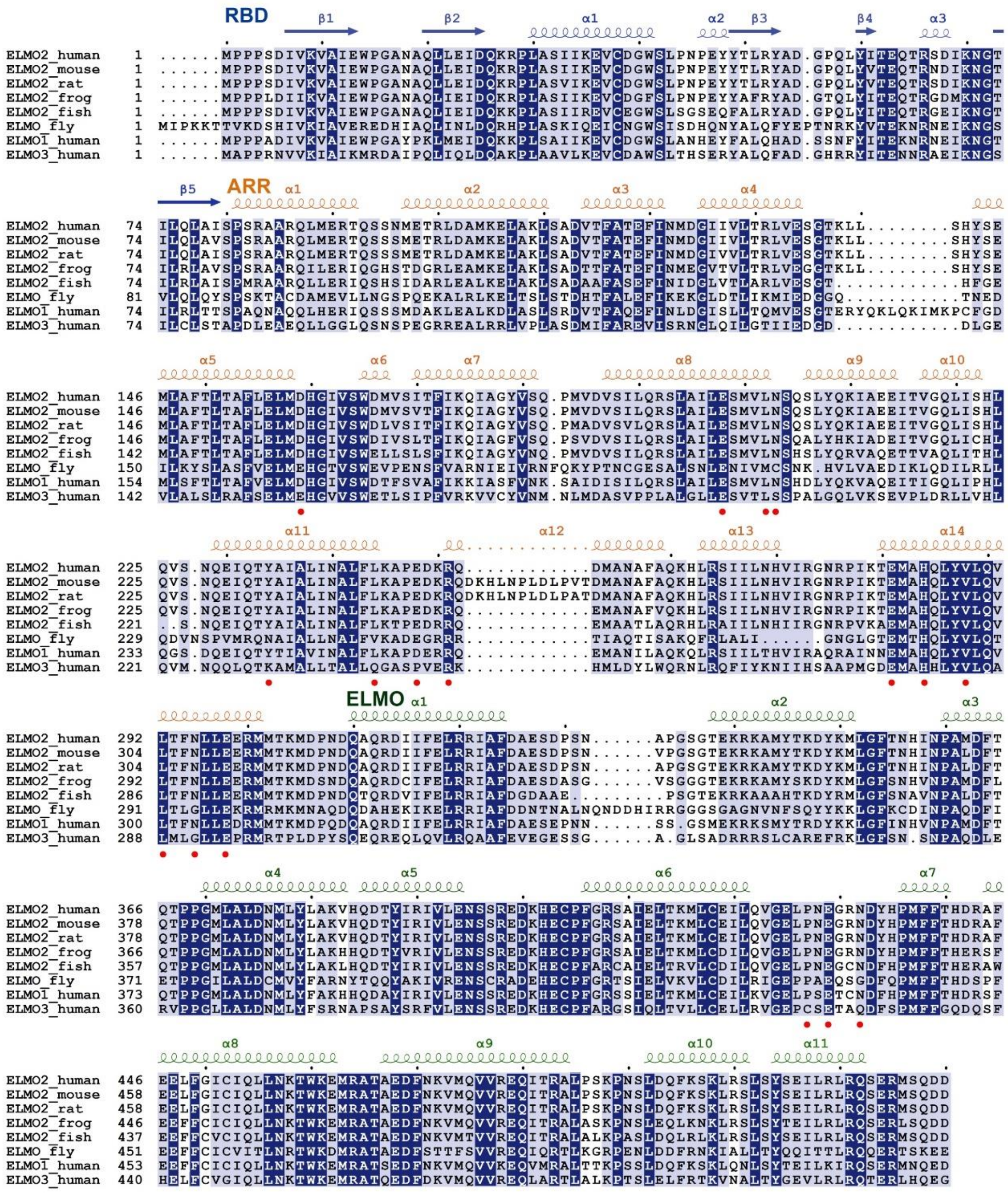


**Supplementary Figure 3 | Sequence alignment of the RAE tandems from the ELMO family members.** In this alignment, residues that are absolutely conserved and highly conserved are highlighted in dark and light blue, respectively. The secondary structural elements are indicated above the alignment and the coloring scheme matches with the structure of the protein shown in Fig. 2a. Residues involved in BAI1 binding are annotated below as red dots.

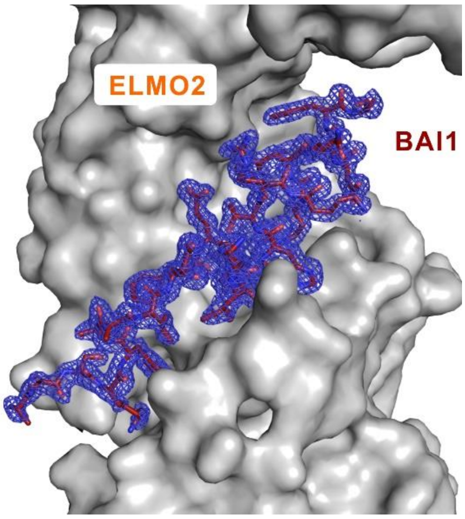


**Supplementary Figure 4 | Electron density of the BAI1 peptide in the ELMO2-RAE/BAI1-EBD complex.** The 2Fo-Fc map was calculated by omitting BAI1 peptide from the final model and contoured at 1.0σ.

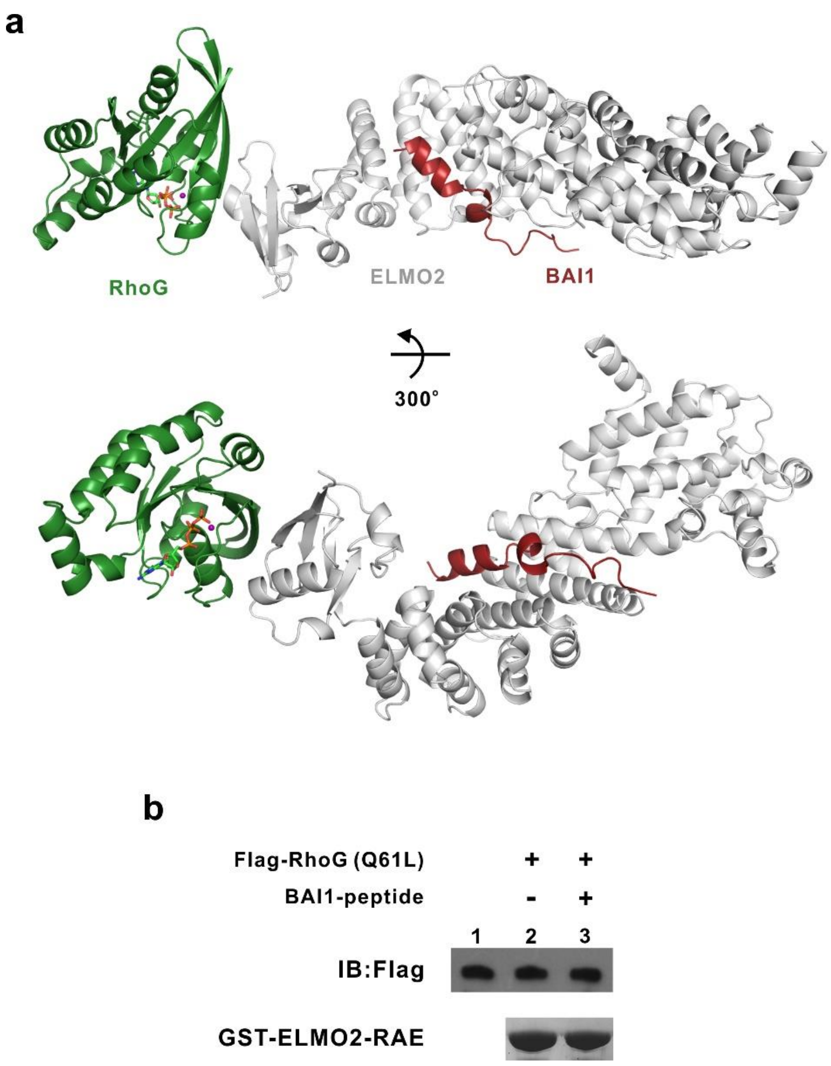


**Supplementary Figure 5 | Binding of BAI1 to ELMO2 didn't affect RhoG/ELMO2 interaction. a,** Homology model of RhoG/ELMO2/BAI1 complex based on the Ras/RalGDS complex structure (PDB code: 1LFD). RhoG, ELMO2-RAE and BAI1 peptide are color in green, gray, and red, respectively. Noted that the binding site of BAI1 on ELMO2 is far away from the RhoG-binding site. **b**, Pull down assays showing that active RhoG bound to ELMO2-RAE effectively with (*lane 3*) or without (*lane 2*) excess amount of the BAI1-EBD peptide. In the assays, the concentrations of GST-ELMO2-RAE and the BAI1-EBD peptide were 20 μM and 160 μM, respectively. *Lane 1*, Flag-RhoG-Q61L input.

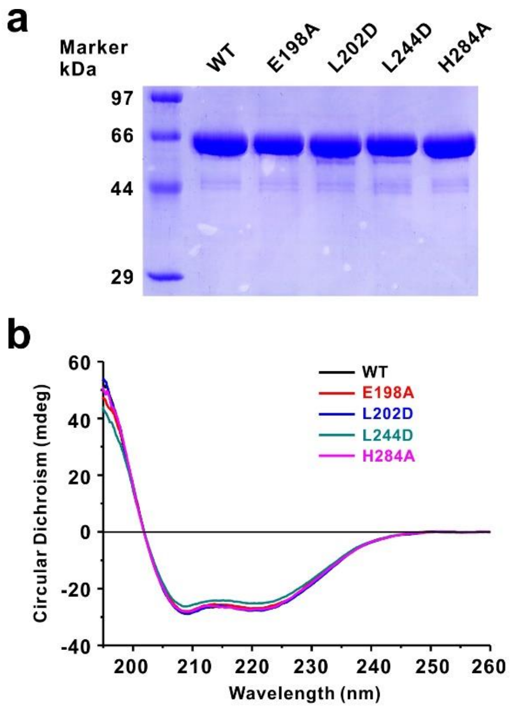


**Supplementary Figure 6 | Mutants of ELMO2-RAE did not induce significant conformational changes of ELMO2-RAE. a,** SDS-PAGE of freshly purified wild-type and mutants of ELMO2-RAE. **b,** Circular dichroism (CD) spectrum of wild-type and mutants of ELMO2-RAE.

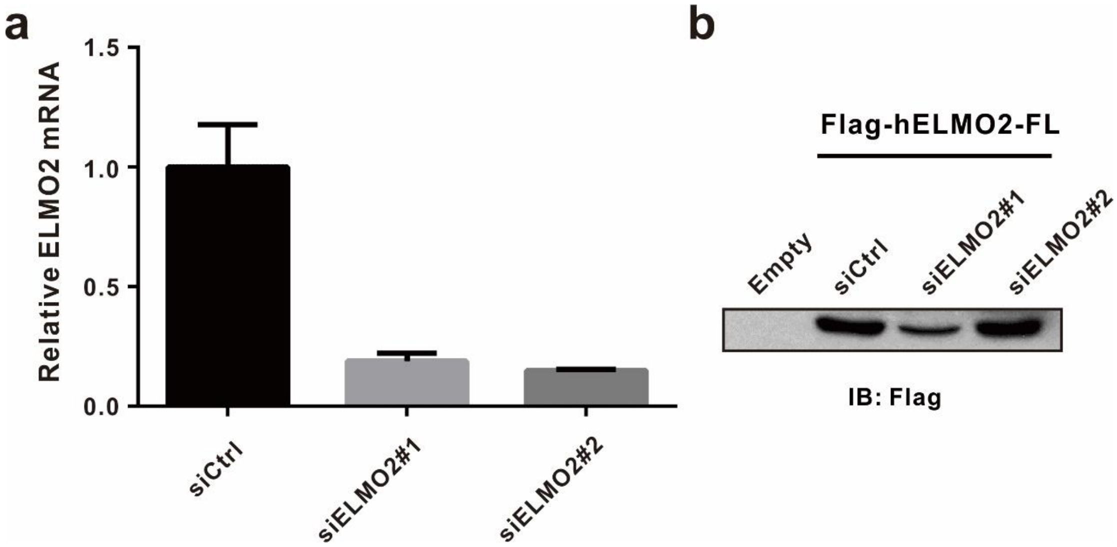


**Supplementary Figure 7 | Real-time Q-RT-PCR amplifications against ELMO2 were performed to confirm specific knockdowns. a**, Two of the siRNAs against ELMO2 significantly reduced the mRNA level in C2C12 cells. Values are mean ± s.d. from three independent experiments. **b**, Western blotting assays showing the expression levels of the exogenous transfected Flag-tagged hEMLO2.

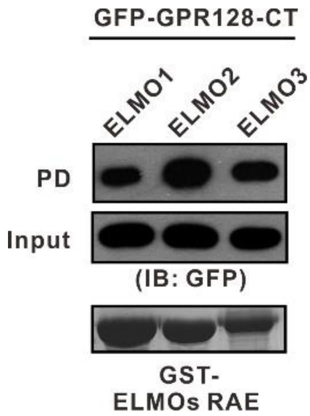


**Supplementary Figure 8 | GST pull down assay showing that the RAE tandem of ELMO1-3 all bound effectively to cytosolic tail of GPR128.** PD, pull down.

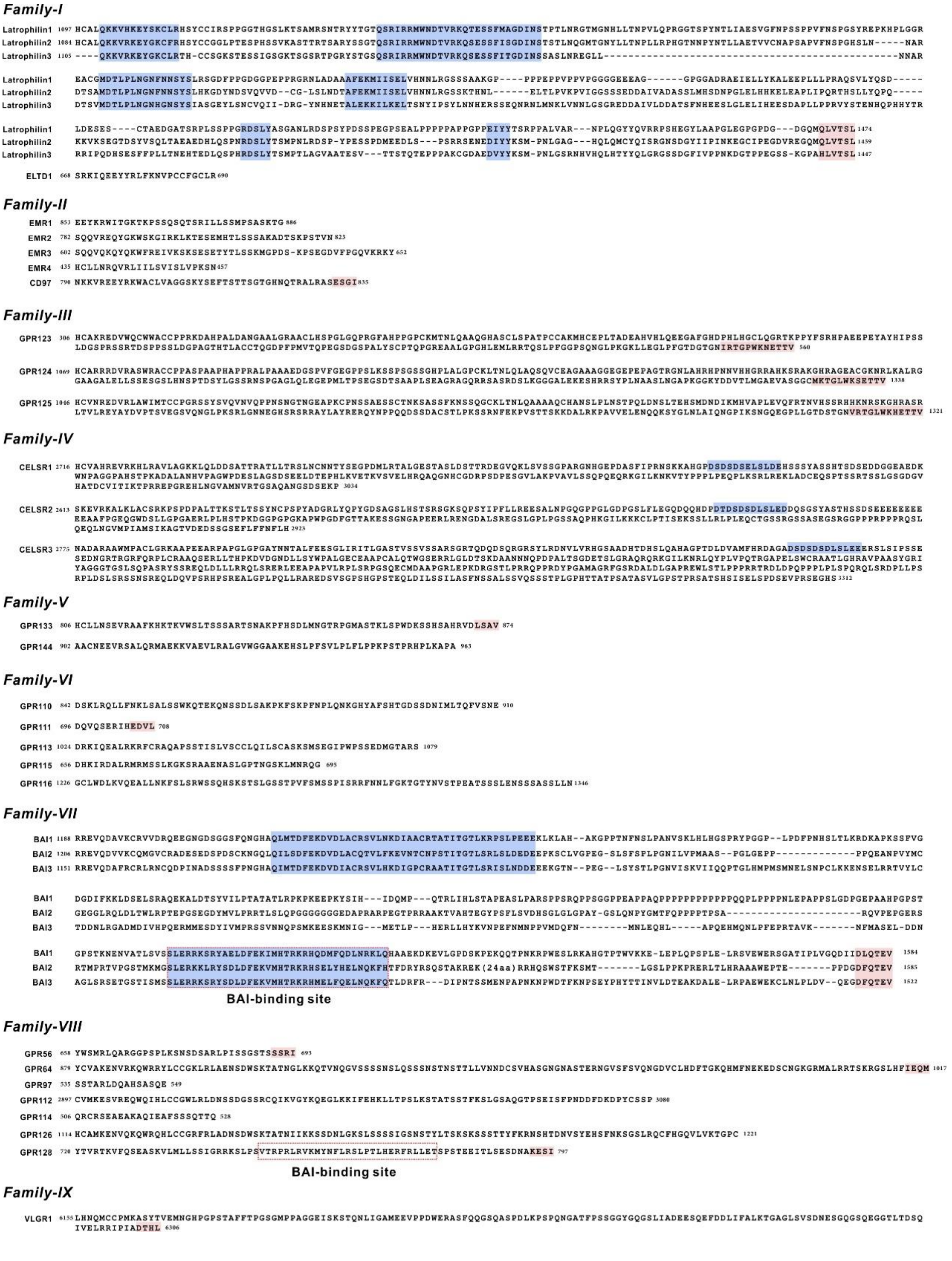

**Supplementary Figure 9 | A comprehensive sequence analysis of the intracellular regions of all adhesion GPCRs.** PDZ-binding motif is highlighted with the pink box; the conserved sequence motif is indicated with the blue box; BAI-binding motif is indicated with the red dashed box.

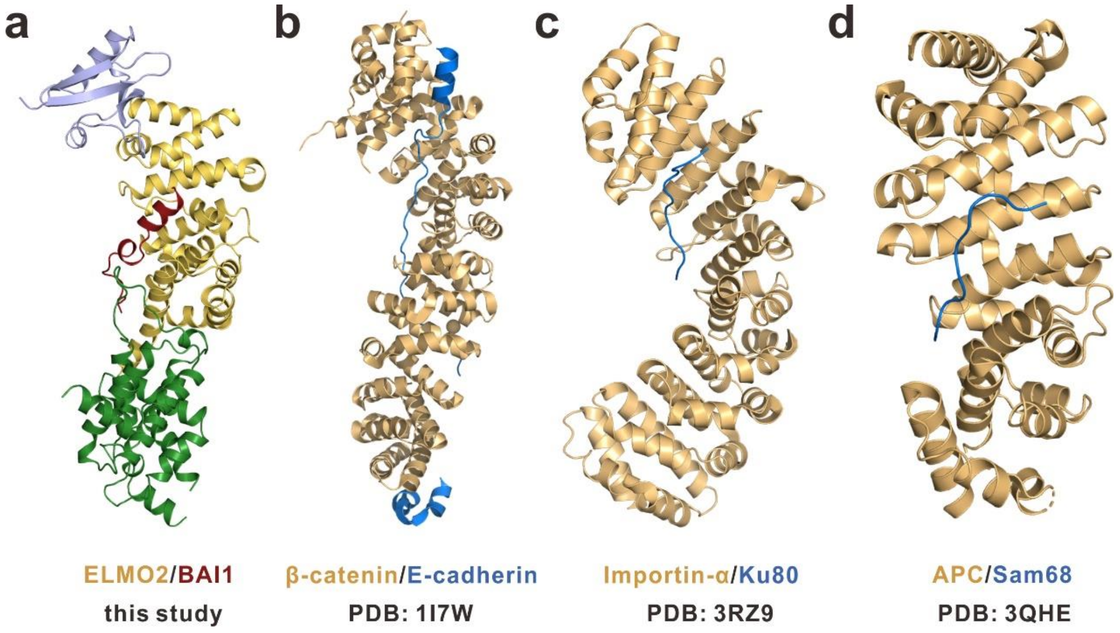


**Supplementary Figure 10 | Gallery of selected ARR/target recognition modes.** Ribbon diagram of complex structure of ELMO2/BAI1 (**a**), β-catenin/E-cadherin (**b**), Importin-α/Ku80 (**c**), and APC/Sam68 (**d**). In this drawing, the ARR domains are colored in orange. The targets of ARRs are colored in blue (BAI1 in red, panel **a**).

## Supplementary Table 1. List of primer sequences used in this study

| Fragments | Primer sequence |
|---|---|
| ELMO2 RBD-ARR-ELMO | UP:GC GGATCC ATGCCGCCTCCGTCTG |
| | DN: CG GCGGCCGC TCA GTCATCCTGACTCATCCTCTC |
| ELMO2 ARR-ELMO | UP: GC GGATCC CCGTCCCGGGCTGCACGCCAG |
| | DN: CG GCGGCCGC TCA GTCATCCTGACTCATCCTCTC |
| ELMO2 RBD-ARR | UP:GC GGATCC ATGCCGCCTCCGTCTG |
| | DN: CG GCGGCCGC TCA GTCCATCTTGGTCATCATCCTTTC |
| ELMO2 ARR | UP: GC GGATCC CCGTCCCGGGCTGCACGCCAG |
| | DN:CG GCGGCCGC TCA GTCCATCTTGGTCATCATCCTTTC |
| ELMO2 ELMO | UP:GC GGATCC CCCAATGACCAGGCTCAAAGGGAC |
| | DN: CG GCGGCCGC TCA GTCATCCTGACTCATCCTCTC |
| BAI1_1431-1582 | UP: GC GGATCC GAGCCTGCACCTCCCAGCCTG |
| | DN: GC CTCGAG TCAGACCTCGGTCTGGAGGTCAATGAT |
| BAI1_1467-1502 | UP: GC GGATCC TCTCTGGAGCGGCGGAAATCAC |
| | DN: GC CTCGAG TCA CTGCAGCTTCCGGTTCAGGTC |
| BAI1_1467-1491 | UP: GC GGATCC TCTCTGGAGCGGCGGAAATCAC |
| | DN: GC CTCGAG TCA TTGGTGTCGCTTCCGTGTGTGCATG |
| BAI2_EBD | UP: GC GGATCC TCC CTT GAG CGA AAG AAG C |
| | DN: GC CTCGAG TCA GTCGAAAGTGTGGAACTTCTGGTT |
| BAI3_EBD | UP: GC GGATCC TCTCTAGAGAGAAGAAAATCG |
| | DN: GC CTCGAG TCAGTCCAAAGTCTGAAACTTCTGATT |
| human BAI1_CT | UP:TACAAGTACTCAGATCTCGAC GGATCCCGT AGAGAGGTCCAGGACGCTG |
| | DN: GATTCGGTGGATCGCTCGAGT GCGGCCGC TCAGACCTCGGTCTGGAGGTCGATG |
| human BAI3_CT | UP:TACAAGTACTCAGATCTCGAC GGATCC CACTGCATTCTTCGGAGAGAG |
| | DN:GATTCGGTGGATCGCTCGAGT GCGGCCGC TTAAACTTCTGTTTGAAAGTCACC |
| GPR128_CT | UP: GC GGATCC ACTGTTCGAACAAAAGTATTTCAG |
| | DN: GC GCGGCCGC TCAAGACAATGATGTTTCCTCAGTC |
