# Supplementary material for: Structure of BAI1/ELMO2 complex reveals an action mechanism of adhesion GPCRs via ELMO family scaffolds
Source: Nat Commun. 2019 Jan 3;10:51. doi: 10.1038/s41467-018-07938-9 (PMC6318265; doi:10.1038/s41467-018-07938-9)
Supplement: Supplementary file 3 — Reporting Summary [file 41467_2018_7938_MOESM3_ESM.pdf]

nature research

Corresponding author(s): Jinwei Zhu

Reporting Summary

Nature Research wishes to improve the reproducibility of the work that we publish. This form provides structure for consistency and transparency in reporting. For further information on Nature Research policies, see Authors & Referees and the Editorial Policy Checklist.

Statistical parameters

When statistical analyses are reported, confirm that the following items are present in the relevant location (e.g. figure legend, table legend, main text, or Methods section).

n/a	Confirmed	
☐	☒	The exact sample size (n) for each experimental group/condition, given as a discrete number and unit of measurement
☐	☒	An indication of whether measurements were taken from distinct samples or whether the same sample was measured repeatedly
☒	☐	The statistical test(s) used AND whether they are one- or two-sided<br>*Only common tests should be described solely by name; describe more complex techniques in the Methods section.*
☒	☐	A description of all covariates tested
☒	☐	A description of any assumptions or corrections, such as tests of normality and adjustment for multiple comparisons
☐	☒	A full description of the statistics including central tendency (e.g. means) or other basic estimates (e.g. regression coefficient) AND variation (e.g. standard deviation) or associated estimates of uncertainty (e.g. confidence intervals)
☐	☒	For null hypothesis testing, the test statistic (e.g. F, t, r) with confidence intervals, effect sizes, degrees of freedom and P value noted<br>*Give P values as exact values whenever suitable.*
☒	☐	For Bayesian analysis, information on the choice of priors and Markov chain Monte Carlo settings
☒	☐	For hierarchical and complex designs, identification of the appropriate level for tests and full reporting of outcomes
☒	☐	Estimates of effect sizes (e.g. Cohen's d, Pearson's r), indicating how they were calculated
☐	☒	Clearly defined error bars<br>*State explicitly what error bars represent (e.g. SD, SE, CI)*

Our web collection on statistics for biologists may be useful.

Software and code

Policy information about availability of computer code

Data collection	No software was used.
Data analysis	Data analysis was carried out in HKL3000 (HKL Research Inc.), Pymol, Image Lab (Bio-Rad) and Origin7.0 (Microcal)

For manuscripts utilizing custom algorithms or software that are central to the research but not yet described in published literature, software must be made available to editors/reviewers upon request. We strongly encourage code deposition in a community repository (e.g. GitHub). See the Nature Research guidelines for submitting code & software for further information.

Data

Policy information about availability of data

All manuscripts must include a data availability statement. This statement should provide the following information, where applicable:

- Accession codes, unique identifiers, or web links for publicly available datasets
- A list of figures that have associated raw data
- A description of any restrictions on data availability

The atomic coordinates of ELMO2-RAE and the ELMO-RAE/BAI1-EBD complex have been deposited to the Protein Data Bank under the accession codes 6IE1 and 6IDX, respectively. Other data are available from the corresponding author upon reasonable request. A reporting summary for this Article is available as a Supplementary Information file. The source data underlying Figs. 1c-e, 4e–f, 5c-d, 6c-d and Supplementary Figs. 1c, 5b and 8 are provided as a Source Data file.

April 2018

Field-specific reporting

Please select the best fit for your research. If you are not sure, read the appropriate sections before making your selection.

☒ Life sciences ☐ Behavioural & social sciences ☐ Ecological, evolutionary & environmental sciences

For a reference copy of the document with all sections, see nature.com/authors/policies/ReportingSummary-flat.pdf

Life sciences study design

All studies must disclose on these points even when the disclosure is negative.

Sample size	No statistical methods were used to determine sample size. The number of biological replicates are indicated in the figure legends.
Data exclusions	No data are excluded.
Replication	All the experimental findings are reproducible.
Randomization	All findings were reproduced in three different experimental replicates.
Blinding	The investigators were blinded to group allocation during data collection.

Reporting for specific materials, systems and methods

Materials & experimental systems

n/a	Involved in the study	
☒	☐	Unique biological materials
☐	☒	Antibodies
☐	☒	Eukaryotic cell lines
☒	☐	Palaeontology
☒	☐	Animals and other organisms
☒	☐	Human research participants

Methods

n/a	Involved in the study	
☒	☐	ChIP-seq
☒	☐	Flow cytometry
☒	☐	MRI-based neuroimaging

Antibodies

Antibodies used	anti-Flag(Sigma; Cat.No.F1804)<br>anti-GFP (Santa Cruze; Cat.No.sc-9996)<br>anti-MHC(DSHB; Cat.No.MF20)<br>Alexa Fluor 488 conjugated donkey anti-mouse secondary antibody (Invitrogen; Cat.No.A-21202)<br>DAPI (Sigma; Cat.No.D9542)
Validation	All the antibodies used in the present study are well verified commercial antibodies.

Eukaryotic cell lines

Policy information about cell lines

Cell line source(s)	HEK 293T cells and C2C12 cells (from ATCC) were used in this study
Authentication	All the cell lines are commercial and authenticated.
Mycoplasma contamination	We confirm that all cell lines tested negative for mycoplasma contamination.
Commonly misidentified lines (See ICLAC register)	No commonly misidentified cell lines were used.
