# Supplementary material for: Structure of BAI1/ELMO2 complex reveals an action mechanism of adhesion GPCRs via ELMO family scaffolds
Source: Nat Commun. 2019 Jan 3;10:51. doi: 10.1038/s41467-018-07938-9 (PMC6318265; doi:10.1038/s41467-018-07938-9)
Supplement: Supplementary file 4 — Source Data [file 41467_2018_7938_MOESM4_ESM.docx]

**Source Data**

**“Structure of BAI1/ELMO2 complex reveals an action mechanism of adhesion GPCRs via ELMO family scaffolds”, Z. Weng et al.**

**Source Data-1**


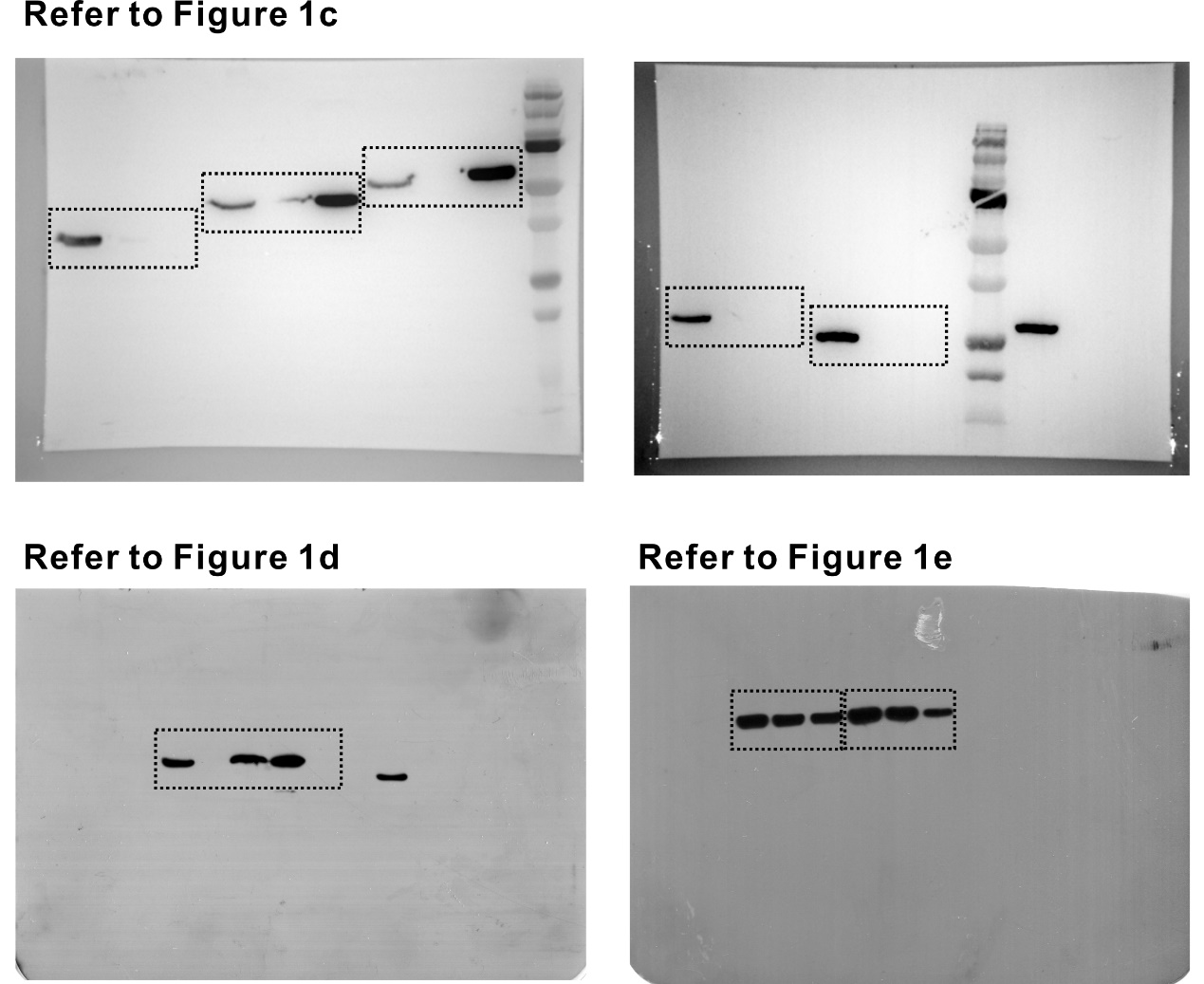


**Source Data-1. Raw images of the western blots used in Figure 1.**

**Source Data-2**


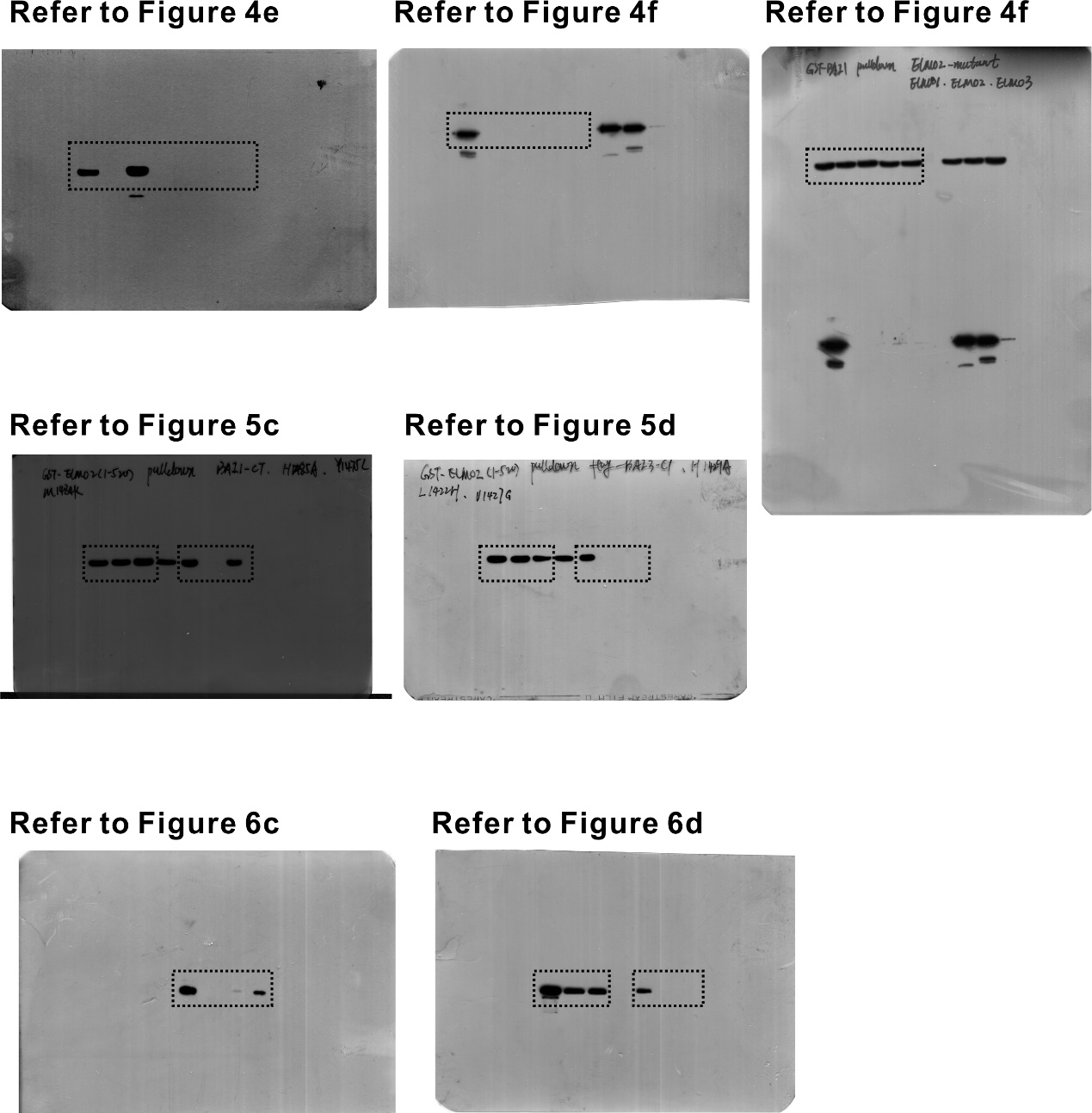


**Source Data-2. Raw images of the western blots used in Figure 4, 5, and 6.**

**Source Data-3**


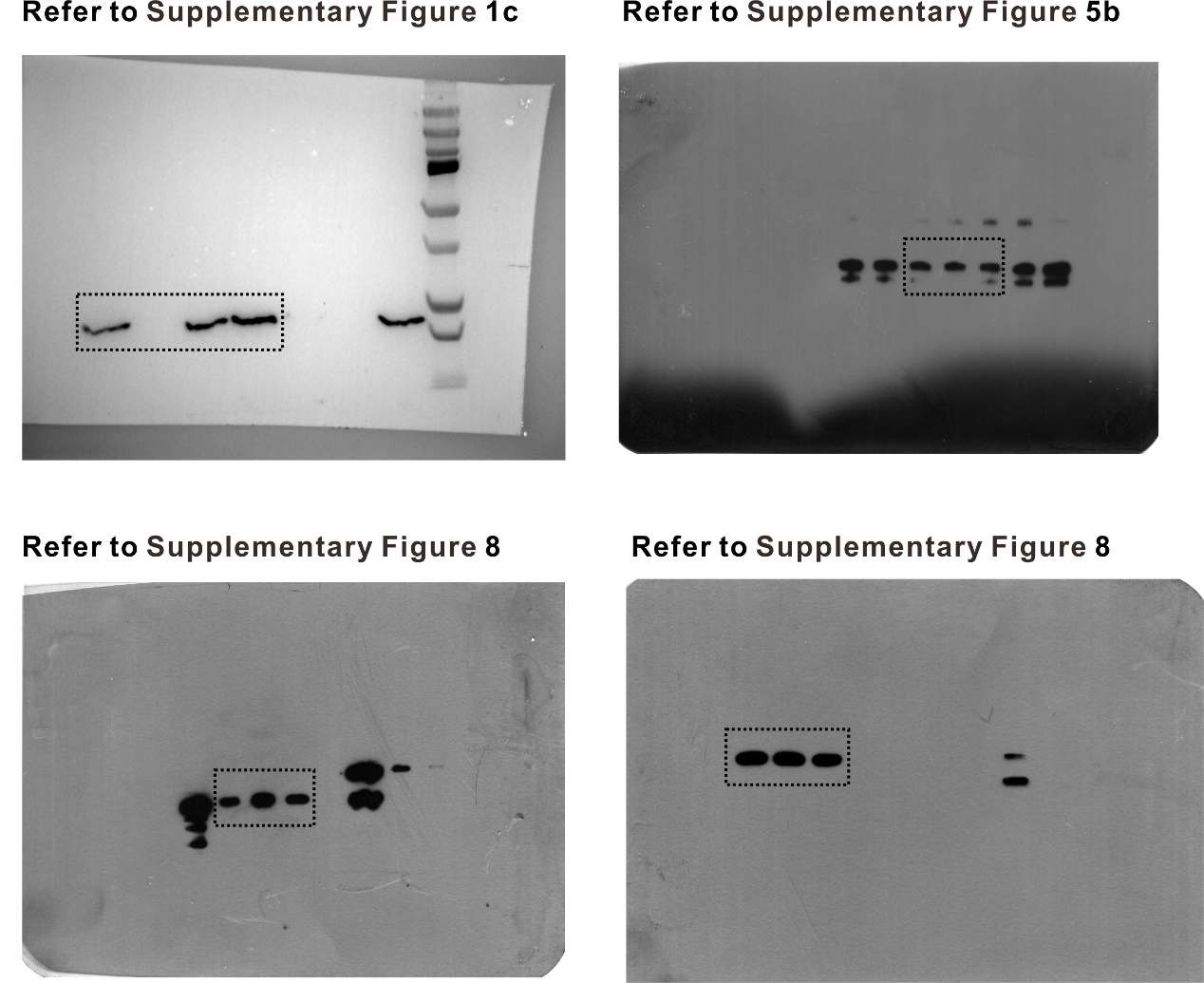


**Source Data-3. Raw images of the western blots used in Supplementary Figure 1c, 5b and 8.**
